# FNDC5/irisin ameliorates bone loss of type 1 diabetes by suppressing endoplasmic reticulum stress‑mediated ferroptosis

**DOI:** 10.1186/s13018-024-04701-3

**Published:** 2024-03-30

**Authors:** Qianqian Dong, Ziqi Han, Mingdong Gao, Limin Tian

**Affiliations:** 1 The First School of Clinical Medicine, Lanzhou University, Lanzhou, 730000 China; 2 Department of Endocrinology, Gansu Provincial Hospital, Lanzhou, 730000 China; 3 Clinical Research Center for Metabolic Disease, Gansu Province, Lanzhou, 730000 China; 4 Department of Pediatrics, Gansu Provincial Hospital, Lanzhou, 730000 China

**Keywords:** Ferroptosis, ER stress, FNDC5/irisin, Type 1 diabetes mellitus, Diabetic osteopathy, Bioinformatics, Osteoblasts

## Abstract

**Background:**

Ferroptosis is known to play a crucial role in diabetic osteopathy. However, key genes and molecular mechanisms remain largely unclear. This study aimed to identify a crucial ferroptosis-related differentially expressed gene (FR-DEG) in diabetic osteopathy and investigate its potential mechanism.

**Methods:**

We identified fibronectin type III domain-containing protein 5 (FNDC5)/irisin as an essential FR-DEG in diabetic osteopathy using the Ferroptosis Database (FerrDb) and GSE189112 dataset. Initially, a diabetic mouse model was induced by intraperitoneal injection of streptozotocin (STZ), followed by intraperitoneal injection of irisin. MC3T3-E1 cells treated with high glucose (HG) were used as an in vitro model. FNDC5 overexpression plasmid was used to explore underlying mechanisms in vitro experiments. Femurs were collected for micro-CT scan, histomorphometry, and immunohistochemical analysis. Peripheral serum was collected for ELISA analysis. Cell viability was assessed using a CCK-8 kit. The levels of glutathione (GSH), malondialdehyde (MDA), iron, reactive oxygen species (ROS), and lipid ROS were detected by the corresponding kits. Mitochondria ultrastructure was observed through transmission electron microscopy (TEM). Finally, mRNA and protein expressions were examined by quantitative real‐time PCR (qRT‐PCR) and western blot analysis.

**Results:**

The expression of FNDC5 was found to be significantly decreased in both in vivo and in vitro models. Treatment with irisin significantly suppressed ferroptosis and improved bone loss. This was demonstrated by reduced lipid peroxidation and iron overload, increased antioxidant capability, as well as the inhibition of the ferroptosis pathway in bone tissues. Furthermore, in vitro studies demonstrated that FNDC5 overexpression significantly improved HG-induced ferroptosis and promoted osteogenesis. Mechanistic investigations revealed that FNDC5 overexpression mitigated ferroptosis in osteoblasts by inhibiting the eukaryotic initiation factor 2 alpha (eIF2α)/activated transcription factor 4 (ATF4)/C/EBP-homologous protein (CHOP) pathway.

**Conclusions:**

Collectively, our study uncovered the important role of FNDC5/irisin in regulating ferroptosis of diabetic osteopathy, which might be a potential therapeutic target.

**Supplementary Information:**

The online version contains supplementary material available at 10.1186/s13018-024-04701-3.

## Introduction

Diabetic osteopathy, a severe complication of type 1 diabetes mellitus (T1DM), is often accompanied by low bone mass and increased skeletal fragility [1, 2]. Compared to healthy individuals, T1DM patients have a higher risk of bone fractures [3]. This not only diminishes patients' quality of life but also imposes a significant economic burden. However, there are no specific drugs for the treatment of diabetic osteopathy. Current therapies are expensive and have undesirable side effects [4, 5]. It is urgent to explore novel drugs and molecular targets for diabetic osteopathy.

It is well-established that type 1 diabetic osteopathy is mainly attributed to impaired bone formation [6]. Several mechanisms, including impaired osteoblast differentiation, apoptosis, metabolic abnormalities, chronic oxidative stress, and inflammation, are associated with bone loss induced by T1DM [7–9]. Notably, as a novel form of regulated cell death [10], ferroptosis has been reported to be involved in osteoporosis [11, 12]. Ferroptosis is featured as iron overload and the accumulation of iron-dependent lipid peroxidation*.* Intracellular iron overload can produce excess amounts of reactive oxygen species (ROS) and induce the production of lipids peroxide, ultimately triggering ferroptosis [13]. On the other hand, the inactivation of glutathione peroxidase 4 (GPX4), inhibition of the cystine-glutamate antiporter system (Xc- system), and depletion of glutathione (GSH) are primarily responsible for the accumulation of lipid peroxides, thus inducing ferroptosis [14]. It is believed that inhibition of ferroptosis could ameliorate osteoporosis. For example, melatonin alleviated diabetic osteoporosis by inhibiting osteoblasts ferroptosis via the Nrf2/HO-1 pathway [15]. Hence, targeting ferroptosis could be a promising therapeutic approach for diabetic osteopathy.

Recent studies suggest that fibronectin type III domain-containing protein 5 (FNDC5) plays an important regulatory role in diabetic osteopathy [16]. FNDC5 is a type I glycosylated transmembrane protein that is expressed in skeletal muscle, bone, heart, and brain [17]. The extracellular portion of FNDC5 is cleaved to produce irisin, which is secreted into the circulation [18]. Since its discovery in 2012, FNDC5/irisin has been reported to maintain bone homeostasis [19]. Dysregulation of FNDC5/irisin is associated with metabolic bone diseases [20]. A recent study showed that patients with T1DM have lower circulating levels of irisin compared to the healthy controls [21]. Similarly, postmenopausal women with osteoporotic hip fractures exhibited lower serum levels of irisin compared with normal control women [22]. The administration of FNDC5/irisin was considered a potential therapeutic approach to improve or treat osteoporosis. Previous results showed that irisin treatment increases bone mass in normal mice, prevents disuse-induced bone loss, and protects against osteoporosis of ovariectomized mice in vivo[23–25]. In vitro studies have demonstrated that irisin treatment enhances osteoblast differentiation and prevents apoptosis of osteocytes [24, 26]. In addition, it inhibits bone resorption and osteoclast differentiation [27]. Considering the important role of irisin, the protective effect and underlying mechanism of FNDC5/irisin on diabetic osteopathy deserves further study.

FNDC5/irisin has been implicated in numerous physiological processes, including energy metabolism, oxidative stress, and autophagy [28]. Recent studies have also shown that FNDC5/irisin regulates ferroptosis in certain diseases, such as renal lung ischemia/reperfusion injury and sepsis-associated encephalopathy[29, 30]. However, the impact of irisin on ferroptosis in diabetic osteopathy remains unknown. We hypothesize that irisin can improve T1DM-induced bone loss by downregulating ferroptosis, which may provide an optimal therapeutic target for diabetic osteopathy. It has recently been reported that the endoplasmic reticulum (ER) stress is closely related to the progression of diabetic osteopathy [31]. Endoplasmic reticulum (ER) stress is a cellular response triggered by the abnormal accumulation of misfolded or unfolded proteins within the ER lumen [32]. The activation of ER stress sensor PERK (protein kinase-like kinase) and its downstream the eukaryotic initiation factor 2 alpha (eIF2α)- activated transcription factor 4 (ATF4)- C/EBP-homologous protein (CHOP) signaling could induce osteoblasts apoptosis [31]. On the other hand, several studies have demonstrated that the activation of the eIF2α-ATF4-CHOP signaling pathway could facilitate the occurrence of ferroptosis [33] and inhibition ER stress pathway could alleviate ferroptosis, thereby ameliorating disease outcome [34]. Besides, FNDC5/irisin has been shown to inhibit ER stress to exert protective effects on some diseases, such as acute pancreatitis and myocardial ischemia/reperfusion injury [35]. However, whether FNDC5/irisin can ameliorate diabetic osteopathy through modulating ER stress‑mediated ferroptosis remains to be further investigated.

This study aimed to investigate the role of FNDC5 in diabetic osteopathy and explore its underlying mechanism in regulating ferroptosis. The study may provide new insights into the prevention and treatment of diabetic osteopathy.

## Materials and methods

### Gene expression profiling acquisition and preprocessing

The expression profiling dataset GSE189112 for bone loss in T1DM was downloaded from the Gene Expression Omnibus (GEO) database (http://www.ncbi.nlm.nih.gov/geo/). Normal tibia tissues of control mice and tibia tissues of T1DM mice were applied to this study. The raw expression values were log 2 transformed, and the log 2 transformed values were used for subsequent analyses.

### Recognition of differentially expressed genes (DEGs)

The "limma" package was used to identify DEGs between control and T1D samples. The *P* < 0.05, |log2fold-change (FC)|> 0.5 were selected as the cut-off thresholds according to the previous method described [36]. At the same time, the cluster heatmaps and volcano plots were executed to visualize the DEGs with "heatmap" and "ggplot2" packages in R.

### Functional enrichment analysis

The R packages "clusterProfiler", "enrichplot", "org.Hs.eg.db", and "ggplot2" were used to perform the Gene Ontology (GO) functional enrichment and Kyoto Encyclopedia of Genes and Genomes (KEGG) pathway analysis of DEGs. A significant filtering criterion for enrichment analysis was set at *P* < 0.05.

### Identification of ferroptosis-related differentially expressed genes (FR-DEGs)

A total of 564 ferroptosis-related genes obtained from the FerrDb [37] (http://www.zhounan.org/ferrdb) were intersected with DEGs of GSE189112 to identify FR-DEGs. Subsequently, a Venn diagram was generated with the online website Jveen (https://jvenn.toulouse.inrae.fr/app/index.html).

### PPI network analysis and identification of hub genes

A protein–protein interaction (PPI) network of FR-DEGs was constructed using the STRING database. The PPI network was identified and visualized using Cytoscape software. The Cytohub plugin in Cytoscape (version 3.8.2, https://cytoscape.org/) was utilized to identify hub genes using the degree method, selecting the top 10 genes.

### Animal models

Special pathogen-free (SPF) C57/BL6 mice (8 weeks old, male, body weight 22.15 ± 0.56 g) were purchased from the animal center of Lanzhou University (Lanzhou, China). All mice were kept under standard conditions (12 h/12hlight–dark cycles; 25 °C temperature), with free access to standard food and water. After a week of acclimation, all mice were randomly divided into the control group (CON) and the streptozotocin (STZ) group. The STZ group mice were intraperitoneally injected with 40 mg/kg STZ (S1030, Sigma Aldrich, St Louis, MO, USA) daily for 5 days according to the Animal Models of Diabetic Complications Consortium (AMDCC) protocol. The control group received equal volumes of vehicle (citrate buffer). Three days after treatment, the fasting blood glucose (FBG) levels were measured from the tail vein using a blood glucose meter. Mice with FBG levels over 16.7 mM were considered T1DM. Afterward, the diabetic mice were randomly separated into the T1DM group and the T1DM + irisin group. In the T1DM + irisin group, each mouse was administered 100 μg/kg recombinant irisin (100–65, Peprotech, East Windsor, NJ, USA) by intraperitoneal injection twice a week. The body weight and blood glucose levels were recorded every 2 weeks after an overnight fast. After 12 weeks of induction, overdose of inhaled isoflurane (R510-22, RWD, Shenzhen, China) was used to euthanize mice. This method is considered acceptable according to AVMA Guidelines for the Euthanasia of Animals [38].

### Micro‐CT scanning

The right femurs were scanned using micro-CT (Nemo NMC-100, PINGSENG Healthcare Inc., Shanghai, China). Regions of interest (ROI) were selected in a 1.8 mm region located 0.5 mm below the distal femur growth plate. Images were reconstructed using the Pingseng Avatar software (version 1.7.0). Furthermore, bone morphometric parameters, including the cortical bone mineral density (Ct. BMD, mg/cm^3^), trabecular bone mineral density (Tb. BMD, mg/cm^3^), the bone volume fraction (BV/TV, %), the trabecular number (Tb. N, 1/mm), trabecular thickness (Tb. Th, mm) and the trabecular separation (Tb. Sp, mm) were quantitatively analyzed.

### Histologic analysis

For histologic analysis, specimens were decalcified in 0.5 M EDTA (G1105, Servecibio, Wuhan, China) for 21 days. Subsequently, the specimens were dehydrated, cleared, paraffin-embedded, and cut into 6 μm sections. Masson staining of femoral sections was performed according to the manufacturer’s instructions using a Masson staining kit (G1006, Servicebio, Wuhan, China). Immunohistochemical analysis of the specimens was conducted using specific antibodies FNDC5 (1:200 dilution, 23,995-1-AP, Proteintech, Wuhan, China). Finally, the sections were photographed using an optical microscope (Leica, Germany).

### ELISA assay

Blood samples were collected according to the instructions of ELISA Kits (CUSABIO, Wuhan, China). Briefly, blood samples were centrifuged at 1000 × g for 15 min at 4 °C. Serum samples were aliquoted and stored at − 80 °C. Plasma was then collected and stored at − 80 °C until assayed. Bone formation markers procollagen I N-terminal propeptide (PINP) and osteocalcin (OCN) were measured by PINP ELISA kit and OCN ELISA kit (CSB-E12775m, CSB-E06917m, CUSABIO, Wuhan, China). The absorbance at 450 nm was detected using a microplate reader (Thermo Scientific, USA).

#### Cell culture and treatment

MC3T3-E1 cells (Procell, Wuhan, China) were cultured in α-MEM (C3060-0500, VivaCell, Shanghai, China) medium supplemented with 10% fetal bovine serum (FBS, FSP500, ExCell Bio). The cells were maintained in a humidified incubator at 37 °C and 5% CO_2_. To mimic T1DM culture conditions, cells were stimulated with varying concentrations of glucose in the culture medium for 48 h. On this basis, the cells were pretreated with GSK2606414 (GSK, 1 μM) (HY-18072, MedChem Express, Monmouth Junction, New Jersey, USA) at the specified concentration for 2 h, and GSK remained in the media throughout the period of glucose stimulation.

#### Cell viability assay

Cell viability was evaluated using a CCK8 Assay kit (40,203, Yeasen Biotechnology, Shanghai, China). Cells were treated with different reagents for the indicated times. 10μL CCK-8 reagent was added at indicated time points. Absorbance at 450 nm was measured with a microplate reader (Thermo Scientific, USA).

#### Real-time quantitative polymerase chain reaction (RT-qPCR)

Total RNAs were extracted from cells and tissues with the TRIzol reagent (9109, Takara, Shiga, Japan). RNA was reverse transcribed with Hifair® III 1st Strand cDNA Synthesis SuperMix for qPCR (11,141, Yeasen Biotechnology, Shanghai, China). The qPCR reactions were performed with 1 × Hieff qPCR SYBR Green Master Mix (Yeasen Biotechnology, Shanghai, China). Specific primers for mRNAs were by Tsingke Biotechnology (Beijing, China). The β-actin forward primer sequence was 5′-AAATCGTGCGTGACATCAAAGA-3′, and the β-actin reverse primer sequence was 5′- GCCATCTCCTGCTCGAAGTC-3′. The FNDC5 forward primer sequence was 5′-ACAGAGCCCAGCCAGTGAGC-3′, and the FNDC5 reverse primer sequence was 5′- GCCCACATGAAGAGGACCACAAC-3′. ACTB (β-actin) was employed as an internal control of mRNAs. The expression levels of genes were calculated via the 2^−ΔΔCt^ method.

#### Western blot analysis

Total proteins of cells and tissues were extracted using RIPA lysate solution (R0010, Solarbio, Beijing, China). Protein quantification was conducted by a BCA Protein Assay Kit (PC0020, Solarbio, Beijing, China). The SDS-PAGE gel was made with resolving gel and stacking gel. The resolving gel was prepared with 1.5 M Tris base at pH 8.8 (T1010, Solarbio, Beijing, China), 10% sodium dodecylsulfate (SDS, S8010, Solarbio, Beijing, China), and 30% acrylamide (A1010, Solarbio, Beijing, China). The stacking gel contained was prepared with 1.0 M Tris base at pH 6.8 (T1020, Solarbio, Beijing, China), 10% SDS, and 30% acrylamide. The proteins were then subjected to electrophoresis using SDS-PAGE gels and transferred to PVDF membranes (00839B, Pall Corporation, Port Washington, NY, USA). The membranes were blocked with 5% nonfat dry milk and incubated with primary and secondary antibodies. Lastly, the membrane was detected with the super electrochemiluminescence (ECL) kit (S6009M, US Everbright, Suzhou, China).

The primary antibodies used included β-actin (1:3000, P60035, Abmart), alkaline phosphatase (ALP, 1:1000, T55421, Abmart, Shanghai, China), CHOP (1:1000 dilution, T56694, Abmart, Shanghai, China), solute carrier family 7 member 11 (SLC7A11, 1:1000 dilution, ab175186, Abcam, Cambridge, MA, USA), runt-related transcription factor 2 (RUNX2,1:1000 dilution, ab236639, Abcam, Cambridge, MA, USA), ferritin heavy chain (FTH, 1:1000 dilution, 3998, Cell Signaling Technology, Danvers, MA, USA), GPX4 (1:1000 dilution, 52,455, Cell Signaling Technology, Danvers, MA, USA), osteocalcin (OCN, 1:1000, DF12303, Affinity, Changzhou, China), eIF2α (1:1000, AF6087, Affinity, Changzhou, China), phosphorylation-eukaryotic initiation factor 2 alpha (p-eIF2α,1:1000, AF3087, Affinity, Changzhou, China), ATF4 (1:1000, AF5416, Affinity), and FNDC5 (1:2000, 23,995-1-AP, Proteintech, Wuhan, China).

#### Measurement of ROS levels and lipid peroxidation levels

Intracellular ROS levels were measured by a reactive oxygen species assay kit (S0033, Beyotime Biotechnology, Shanghai, China). Intracellular lipid peroxidation levels were determined by the C11-BODIPY 581/591 fluorescent probe (D3861, Invitrogen, Carlsbad, CA, USA). Firstly, cells were seeded in 24-well plates and treated with specific chemicals for the indicated times. Then the fluorescent probe DCFH-DA or C11-BODIPY581/591 was added to stain the cells. Finally, images were captured with a fluorescence microscope (Olympus, Japan).

#### Measurement of GSH, MDA, and total iron content

GSH is an important antioxidant, and the depletion of GSH can trigger ferroptosis [39]. Malondialdehyde (MDA) is an end-product of lipid peroxides, which is used to assess ferroptosis [40]. The cellular or tissue GSH, MDA content, tissue iron levels and serum iron levels were detected using the reduced glutathione (GSH) assay kit (A006-2-1, Nanjing Jiancheng Bioengineering Institute, Nanjing, China), the malondialdehyde (MDA) detection kit (S0131, Beyotime Biotechnology, Shanghai, China), the tissue iron assay kit (A039-2-1) and the serum iron assay kit (A039-1-1, Nanjing Jiancheng Bioengineering Institute, Nanjing, China) following the manufacturer’s instructions. The concentrations of GSH and MDA were determined by measuring the absorbance at 405 nm and 530 nm. The values for the levels of tissue and serum iron were examined at a wavelength of 520 nm.

#### Transmission Electron Microscope (TEM)

The ultrastructure of mitochondria was observed by TEM. First, cells were collected and fixed in 0.01 MM PBS-buffered 2.5% glutaraldehyde (G1102, Servicebio, Wuhan, China). They were then post-fixed in 1% osmic acid (18,456, Ted Pella, Altadena, CA, USA). Subsequently, samples were dehydrated, embedded in resin, and cut into sections of ∼60 nm. The sections were then dyed with 2% uranyl acetate (02624-AB, SPI, West Chester, PA, USA) and observed with a HITACHI HT7800 electron microscope (Hitachi, Japan).

#### Overexpression of FNDC5

When the cell density reached 70–80% confluence on a 6-well plate, the cells were transfected with an overexpression plasmid of FNDC5 by Lipo8000 transfection reagent (C0533, Byotime Biotechnology, Shanghai, China) according to the manufacturer’s instructions. Cells transfected with an empty plasmid vector were used as the negative control (NC).

#### Alizarin red staining assay and ALP activity test

To induce osteoblast differentiation, MC3T3-E1 cells were treated with osteogenic medium (α-MEM containing 10% FBS, 100 μg/ml ascorbic acid (A8960, Sigma, Sigma-Aldrich, St Louis, MO, USA), 10 mM β-glycerophosphate (G9422, Sigma, Sigma-Aldrich, St Louis, MO, USA), and 10 mM dexamethasone (S4028, Huston, TX, USA) for 14 days. After induction, the cells were washed with PBS and fixed with 4% paraformaldehyde for 30 min. Alizarin red staining was performed using 0.1% Alizarin Red S (G8550, Solarbio, Beijing, China) solution for 30 min. The mineralized nodules were observed by a microscope after washing with ddH2O. The alkaline phosphatase (ALP) activity test was performed using the ALP test kit (A059, Nanjing Jiancheng, Nanjing, China) according to the manufacturer’s instructions.

#### Statistical analysis

Data were expressed as mean ± standard deviation (SD) of at least three independent experiments for the studies. The SPSS 22.0 (SPSS INC, USA) and GraphPad Prism 9.3.1 (GraphPad, USA) were used to analyze experimental data. The Shapiro–Wilk test was performed to evaluate whether the data was normally distributed. Levene’s test was performed to analyze the homogeneity of variances. Statistical analysis was carried out using Student’s t-test between two groups and analysis of variance (ANOVA) among multiple groups in accordance with data normal distribution and homogeneity of variances. The difference was considered statistically significant if *P* < 0.05.

## Results

### Identification of DEGs in T1DM-induced bone loss

**Fig. 1:**
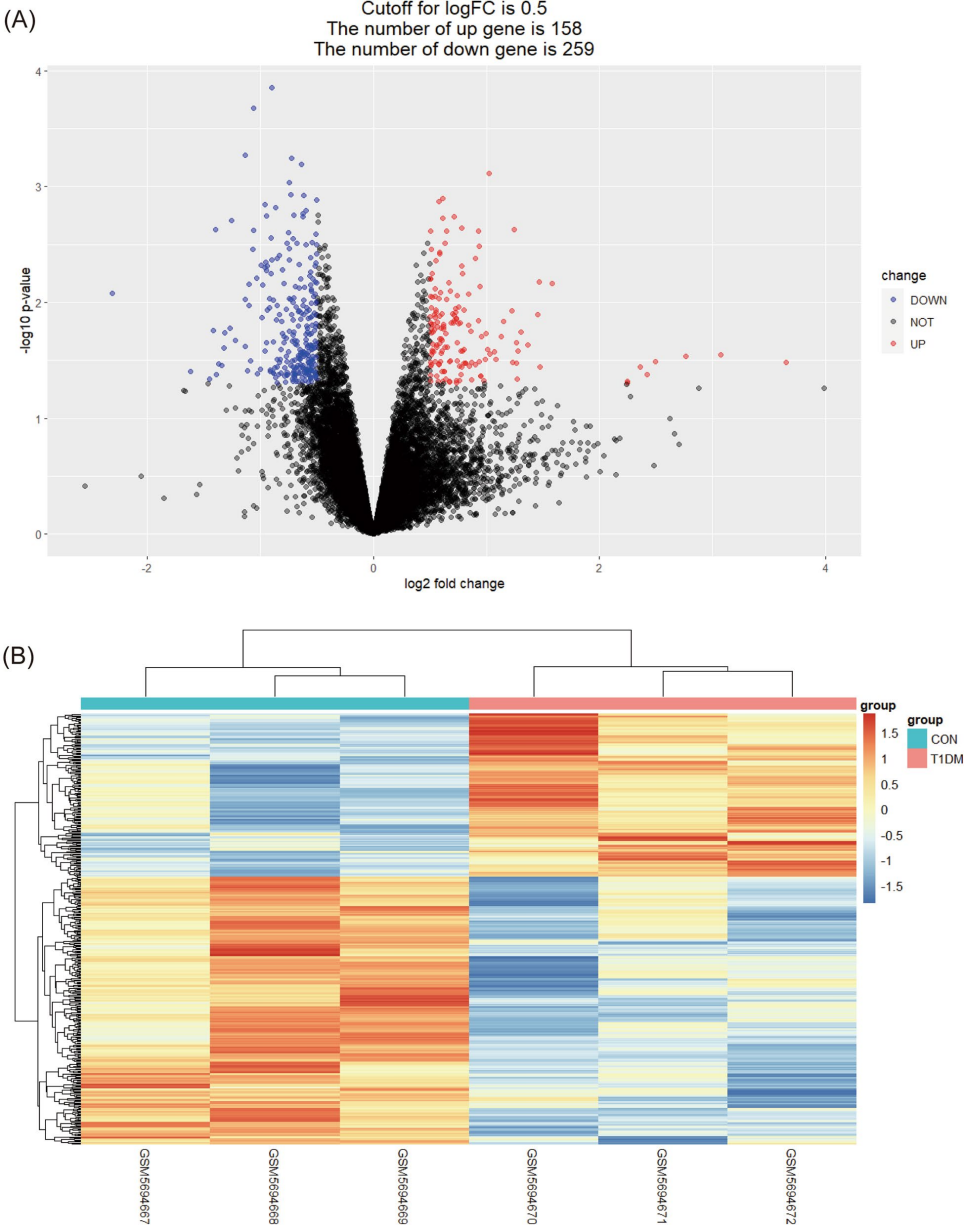
Differentially expressed genes identification. (**A**) Volcano plot corresponding to the GSE189112 dataset. (**B**) Heat map corresponding to the GSE189112 dataset

The GSE189112 gene expression dataset was downloaded and analyzed from the GEO database. 3 normal tibia samples and 3 tibia samples of diabetic mice were used for analysis. A total of 417 DEGs were obtained by filtering at *P* < 0.05 and log2FC > 0.5, respectively. The results of DEGs are shown in the volcano plot and the heatmap and (Fig. 1A, B).

### FNDC5 was identified as an essential FR-DEG in diabetic osteopathy.

**Fig. 2:**
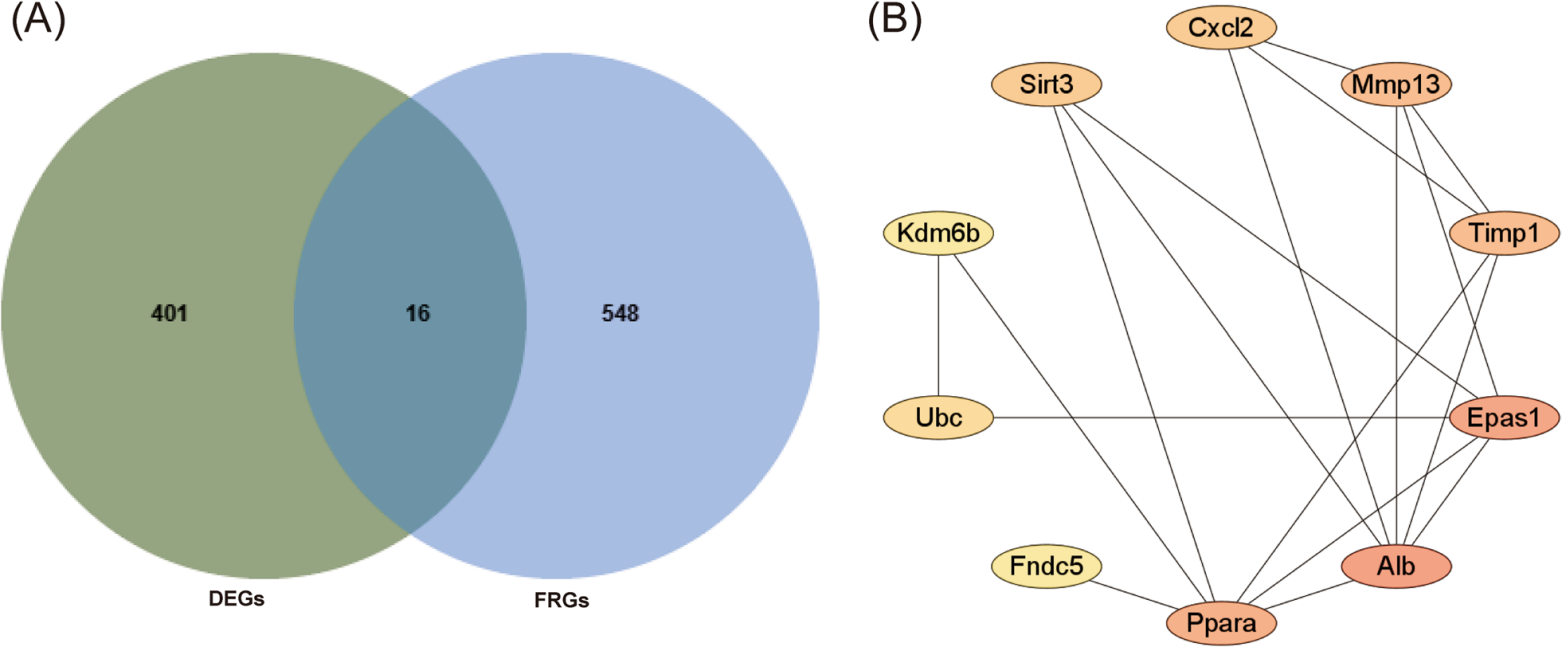
Identification of the ferroptosis-related genes in diabetic osteopathy. (**A**) Venn diagrams of FRGs. (**B**) Cluster plots represent the top 10 hub FRGs identified by cytoHubba

GO and KEGG analysis were conducted to identify the biological functions of DEGs. The results (Additional file 1: Fig. S1A, B) showed that the DEGs were mainly enriched in oxidative phosphorylation and metabolism signaling pathways, and these are related to ferroptosis. Next, we downloaded FRGs from FerrDb and intersected with DEGs to obtain 16 FR-DEGs associated with diabetic osteopathy (Fig. 2A). To further determine the more sensitive candidate genes, we utilized the cytohubba plug-in to obtain the top 10 hub genes, which included FNDC5, ALB, PPARα, CXCL2, MMP13, TIMP1, EPAS1, UBC, KDM6B, SIRT3 (Fig. 2B). Based on the logFC, *P* Value of FR-DEGs (Table 1), and their reported biological functions in literature, we selected FNDC5 as the candidate gene.

**Table 1:** *P* value and log FC of the top 10 hub FR-DEGs

Gene	*P* value	log FC
UBC	0.00152	− 0.86218
FNDC5	0.00196	− 1.25561
ALB	0.00837	− 2.31036
PPARα	0.01066	− 1.09908
KDM6B	0.01314	− 0.54503
CXCL2	0.02236	0.73879
TIMP1	0.02938	2.76520
SIRT3	0.02980	− 0.51101
MMP13	0.03280	3.64719
EPAS1	0.03286	− 0.57721

### FNDC5 expression was decreased in bone tissues of type 1 diabetic mice

**Fig. 3:**
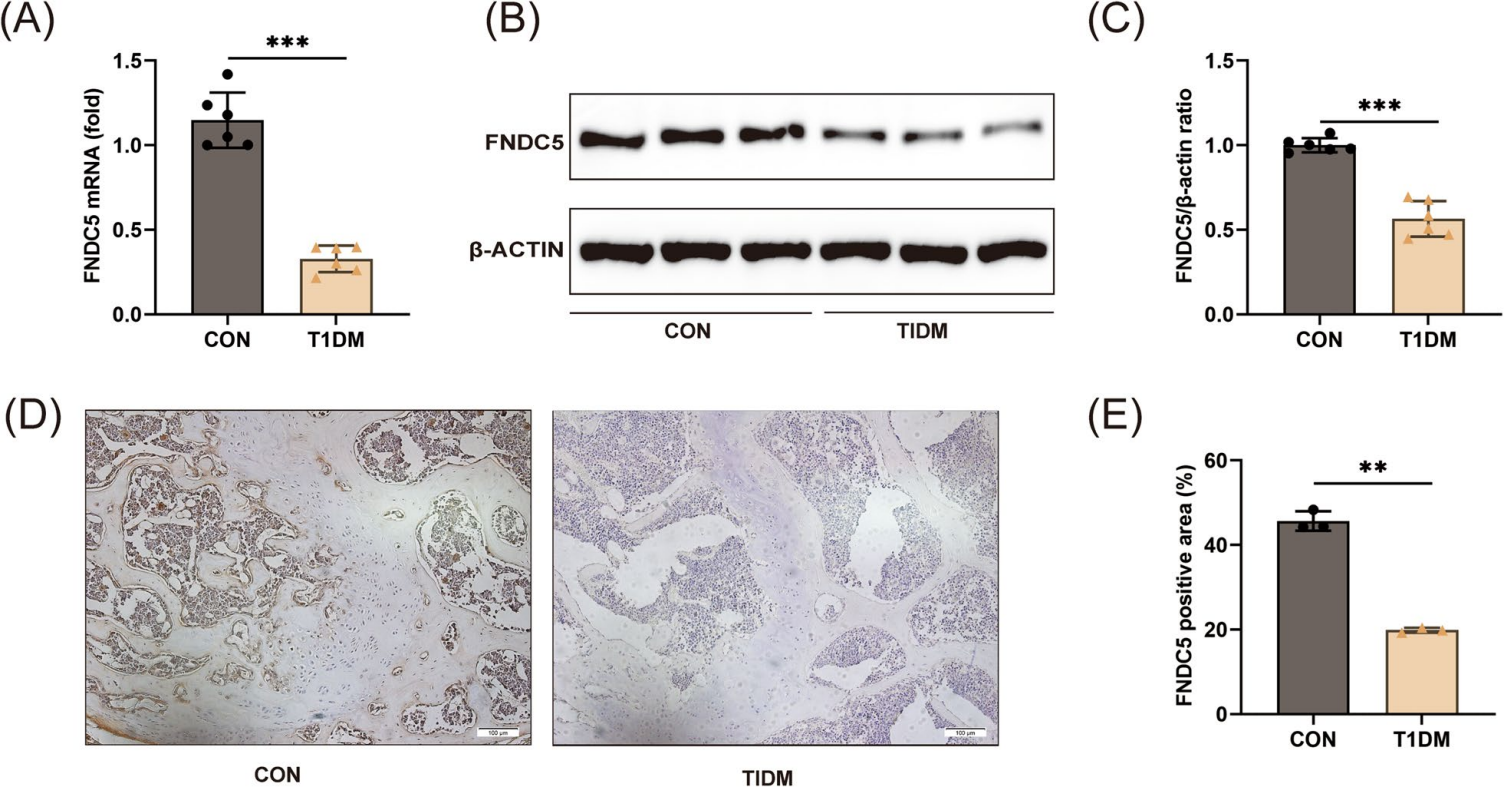
FNDC5 was decreased in the bone tissues of diabetic mice. (**A**) Relative mRNA level of FNDC5 in the bone tissues. n = 6 per group. (**B**, **C**) Relative protein level of FNDC5 in the bone tissues. n = 6 per group. (**D**, **E**) Immunohistochemical staining of FNDC5 in the bone tissues. n = 3 per group. Scale bar: 100 μm. **P < 0.01, ***P < 0.001 versus the CON group

To detect the expression level of FNDC5, we first established an STZ-induced diabetic mouse model. The results showed a significant decrease in both mRNA (Fig. 3A) and protein levels of FNDC5 in the bone tissues of the T1DM group (Fig. 3B, C). Additionally, immunohistochemistry results demonstrated a lower protein expression of FNDC5 in the bone tissues of the T1DM group compared to normal tissue (Fig. 3D, E). These findings are consistent with the results obtained from the bioinformatics analysis.

### FNDC5/irisin treatment improved bone loss of T1DM mice

**Fig. 4:**
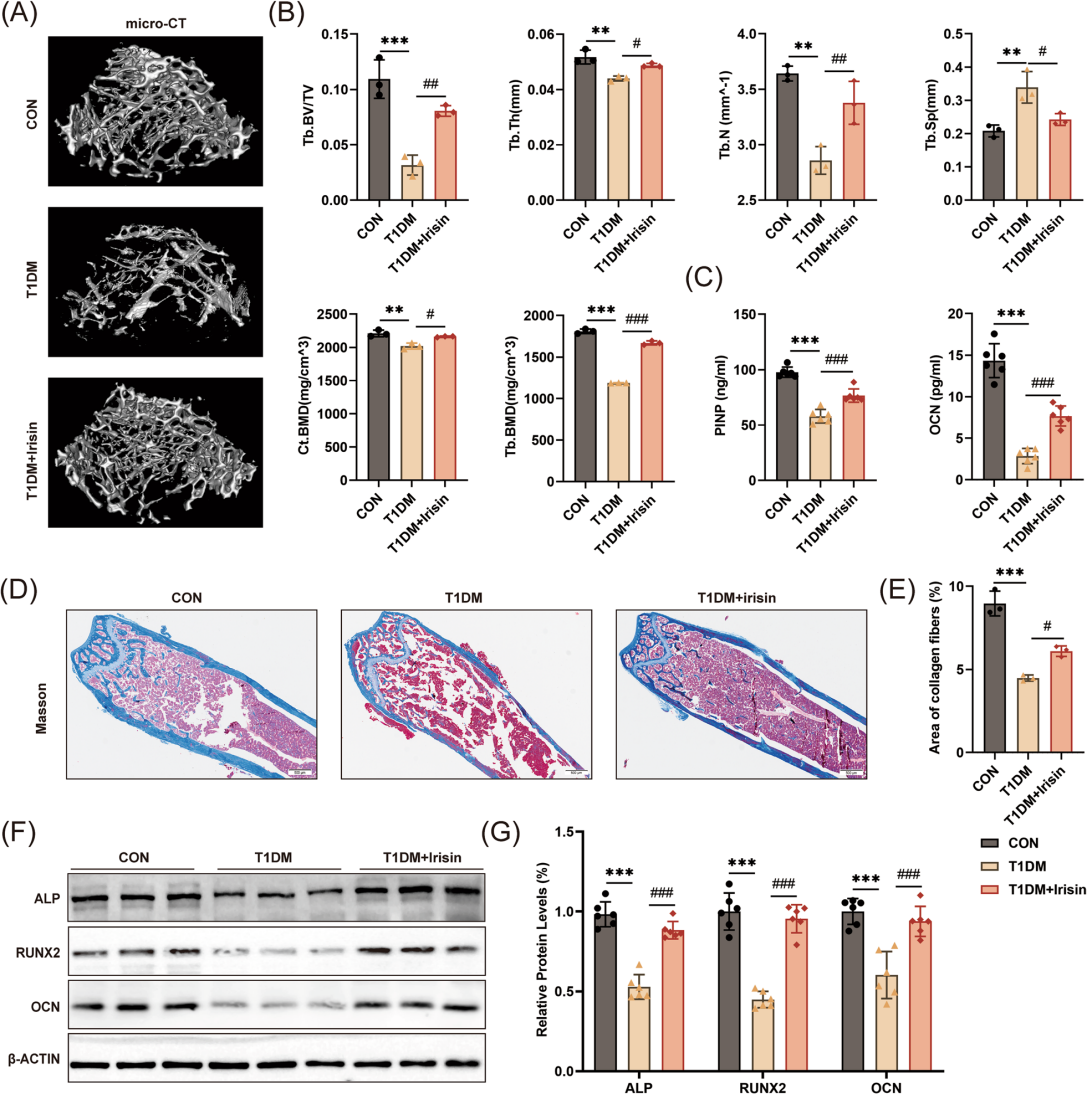
FNDC5/irisin treatment improved bone loss of diabetic mice. (**A**) Micro-CT analysis of the distal femur region and (**B**) the trabecular bone parameters (n = 3). (**C**) Serum concentrations of PINP and OCN (n = 6). (**D**, **E**) Representative Masson-stained images of the distal femur (scale: 500 μm, n = 3). (**F**, **G**) Protein levels of ALP, OCN, and RUNX2 (n = 6). **P < 0.01, ***P < 0.001 versus the CON group. ^#^*P* < 0.05, ^##^*P* < 0.01, ^###^*P* < 0.001 versus the T1DM group

To explore the important role of FNDC5/irisin in diabetic osteopathy, recombinant irisin was infused into diabetic mice via intraperitoneal injection. The findings revealed that the diabetic mice exhibited significantly elevated FBG levels and decreased body weights compared to the control mice. In addition, there were no significant changes in blood glucose levels and body weights after treatment with irisin (Additional file 1: Fig. S2A, B). Next, micro-CT was performed to assess the extent of T1DM-induced bone deficits (Fig. 4A). Compared to the control group, T1DM mice had significantly lower Ct. BMD, Tb. BMD, BV/TV, Tb. N, Tb. Th, as well as a higher value of Tb. Sp (Fig. 4B). Additionally, the serum levels of PINP and OCN, representative bone formation markers, were significantly decreased in the T1DM group compared to the control group (Fig. 4C). The results of Masson staining showed that the bone structure became sparse, and the collagen fiber content was significantly decreased in the T1DM group (Fig. 4D, E). Meanwhile, the results of western blot analysis also showed that the expressions of OCN, RUNX2, and ALP were markedly decreased in the T1DM group compared to the control group (Fig. 4F, G). Notably, these abnormal changes were significantly relieved in the irisin-treated group compared to the T1DM group. Our results indicated that FNDC5/irisin treatment improved bone loss of T1DM mice.

### FNDC5/irisin treatment inhibited ferroptosis of diabetic osteopathy

**Fig. 5:**
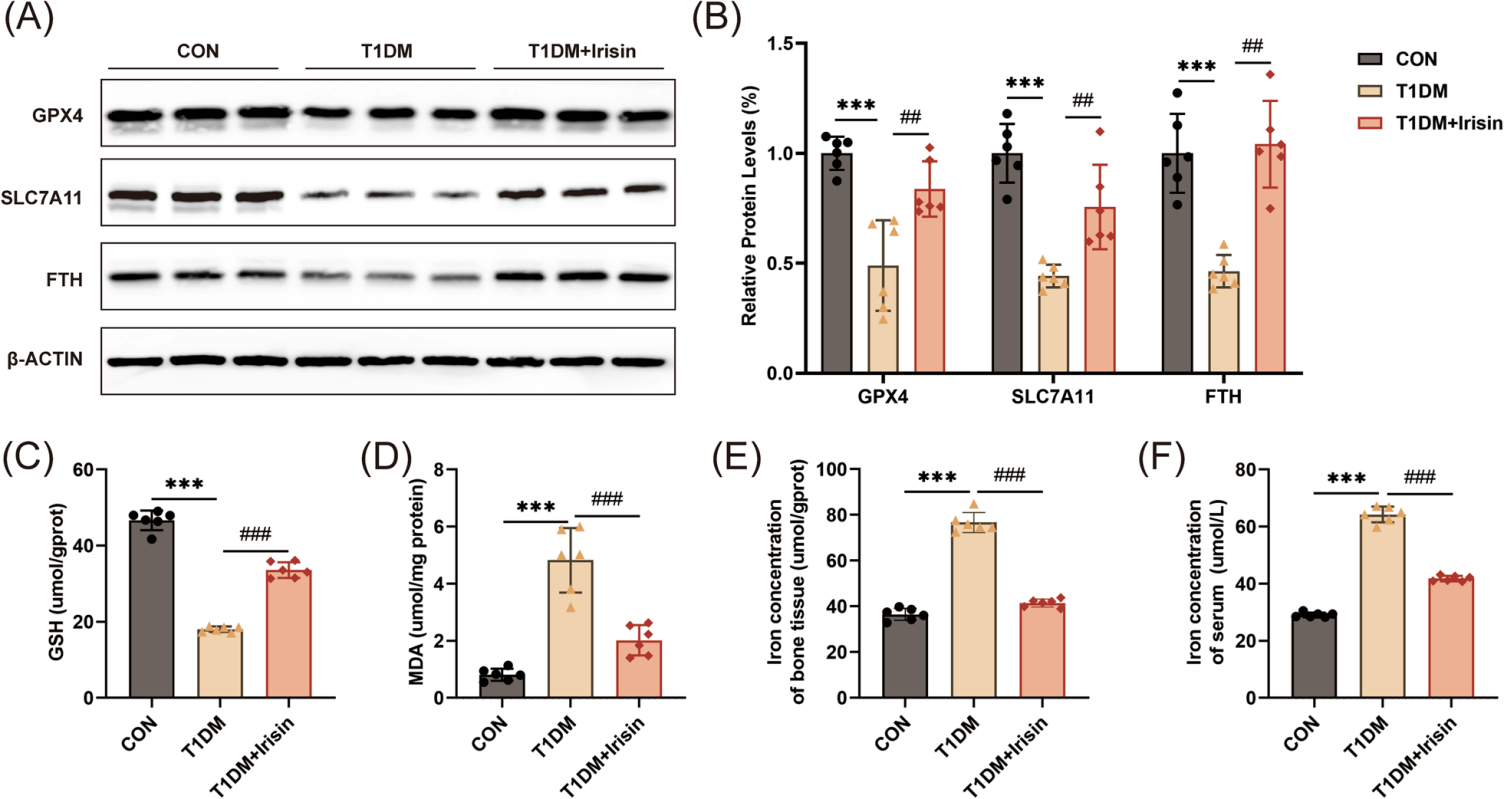
FNDC5/irisin treatment improved ferroptosis of bone tissues in diabetic mice. (**A**, **B**) Protein levels of GPX4, SLC7A11, and FTH (n = 6). (**C**) GSH levels, (**D**) MDA content, (**E**) serum iron levels, and (**F**) tissue iron levels were measured (n = 6). ***P < 0.001 versus the CON group. ^##^*P* < 0.01, ^###^*P* < 0.001 versus the T1DM group

We further explored the effects of FNDC5/irisin on ferroptosis. The results showed that the expressions of GPX4, SLC7A11, and FTH (the classic ferroptosis marker proteins) were reduced in T1DM mice (Fig. 5A, B). The levels of GSH (Fig. 5C) and MDA (Fig. 5D) were significantly decreased in the T1DM group. The iron content in serum and bone tissues was significantly increased in the T1DM group (Fig. 5E, F). The above results implicated that ferroptosis was induced in the bone tissues of diabetic mice. After irisin treatment, ferroptosis related indicators were improved. In conclusion, our findings demonstrate that FNDC5/irisin could improve diabetic osteopathy by inhibiting ferroptosis of bone tissues.

### Overexpression of FNDC5 inhibited HG-induced ferroptosis in MC3T3-E1 cells

**Fig. 6:**
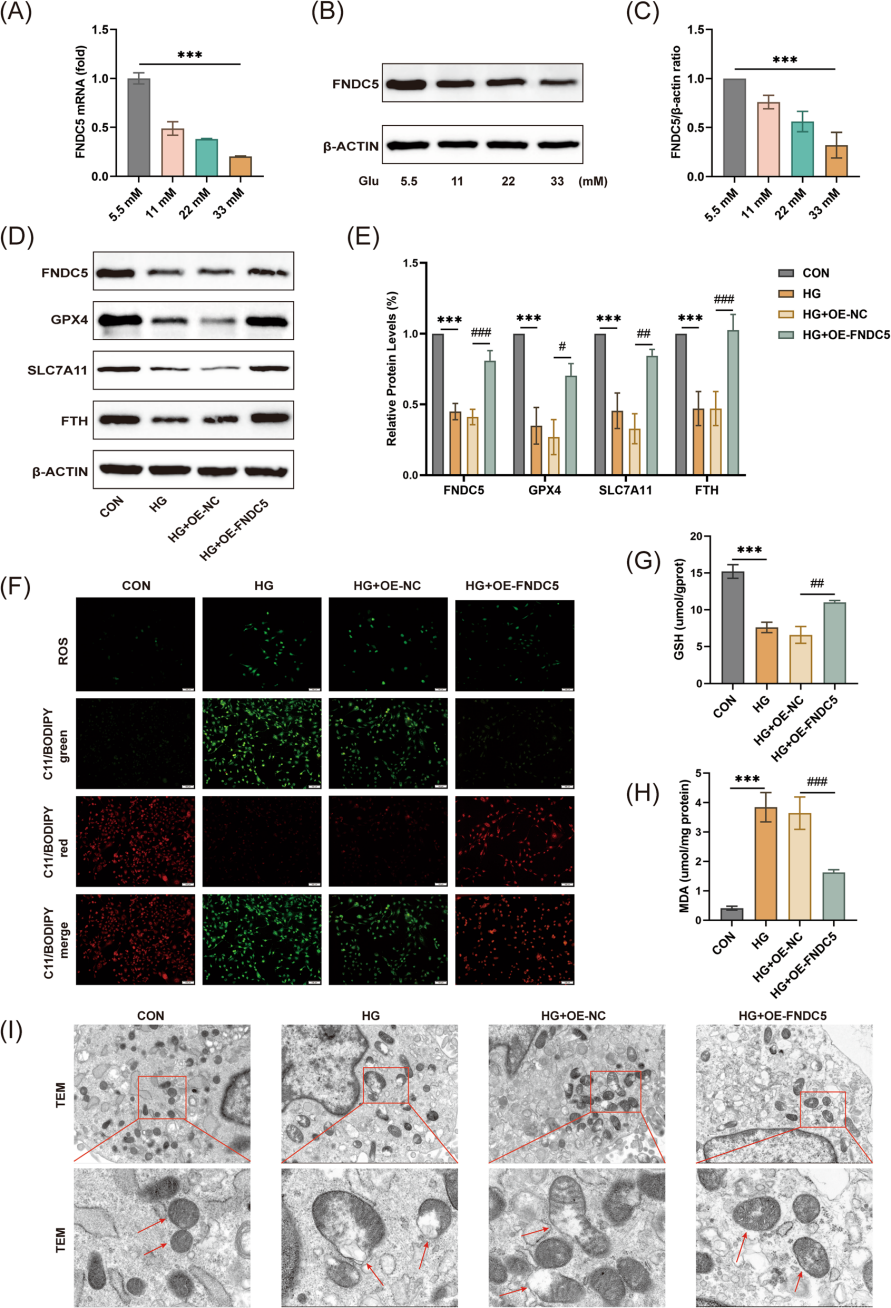
FNDC5 overexpression improved ferroptosis in HG-induced MC3T3-C1 cells. MC3T3-E1 cells were cultured with various glucose concentrations (5.5, 11, 22, or 33 mM). (**A**) Relative mRNA level of FNDC5. (**B**, **C**) Protein level of FNDC5. ***P < 0.001 versus the 5.5 mm group. The cells were treated with HG with or without FNDC5 overexpression. (**D**, **E**) Protein levels of FNDC5, GPX4, SLC7A11, and FTH. (**F**) Representative images of the DCFH-DA staining (scale: 100 μm), and C11-BODIPY staining (scale bar: 100 μm). Red images represented nonoxidized lipids, while green images represented oxidized lipids. (**G**) GSH content and (**H**) MDA levels were measured. (**I**) Representative images of TEM (scale bar: 2.0 μm, 0.5 μm). Red arrowheads point to the mitochondria. OE-NC means negative control, OE-FNDC5 means overexpression of FNDC5. ***P < 0.001 versus the con group, ^##^*P* < 0.01, ^###^*P* < 0.001 versus the HG group

Osteoblasts are key bone metabolism cells that play a significant role in the bone formation of diabetic osteopathy. Therefore, we further explore the underlying mechanism of FNDC5/irisin in inhibiting ferroptosis in MC3T3-E1 cells. Firstly, in vitro data demonstrated that high glucose (33 mM) induced ferroptosis (Additional file 1: Fig. S3) and inhibited FNDC5 expression (Fig. 6A–C). To verify the role of FNDC5 in HG-induced ferroptosis, we transiently overexpressed FNDC5 in MC3T3-E1 cells (Additional file 1: Fig. S4A-C). The results showed that overexpression of FNDC5 significantly reversed the expression of GPX4, SLC7A11, and FTH (Fig. 6D, E). Moreover, FNDC5 overexpression rescued the excessive production of ROS and accumulation of lipid peroxidation (Fig. 6F), restored GSH levels (Fig. 6G), and reduced MDA content (Fig. 6H) induced by HG. TEM was usually used to observe the changes in the morphology of the mitochondria. The TEM images showed that the outer mitochondrial membrane of cells in the HG group had ruptured, and the mitochondrial cristae had decreased or disappeared compared to the control group (Fig. 6I). These are typical cell morphological features of ferroptosis. FNDC5 overexpression improved the abnormal mitochondrial ultrastructure compared with the HG group. In conclusion, these results revealed that FNDC5/irisin has an inhibitory effect on HG-induced ferroptosis of MC3T3-E1 cells.

### FNDC5 overexpression improved HG-induced osteogenic differentiation in MC3T3-E1 cells

**Fig. 7:**
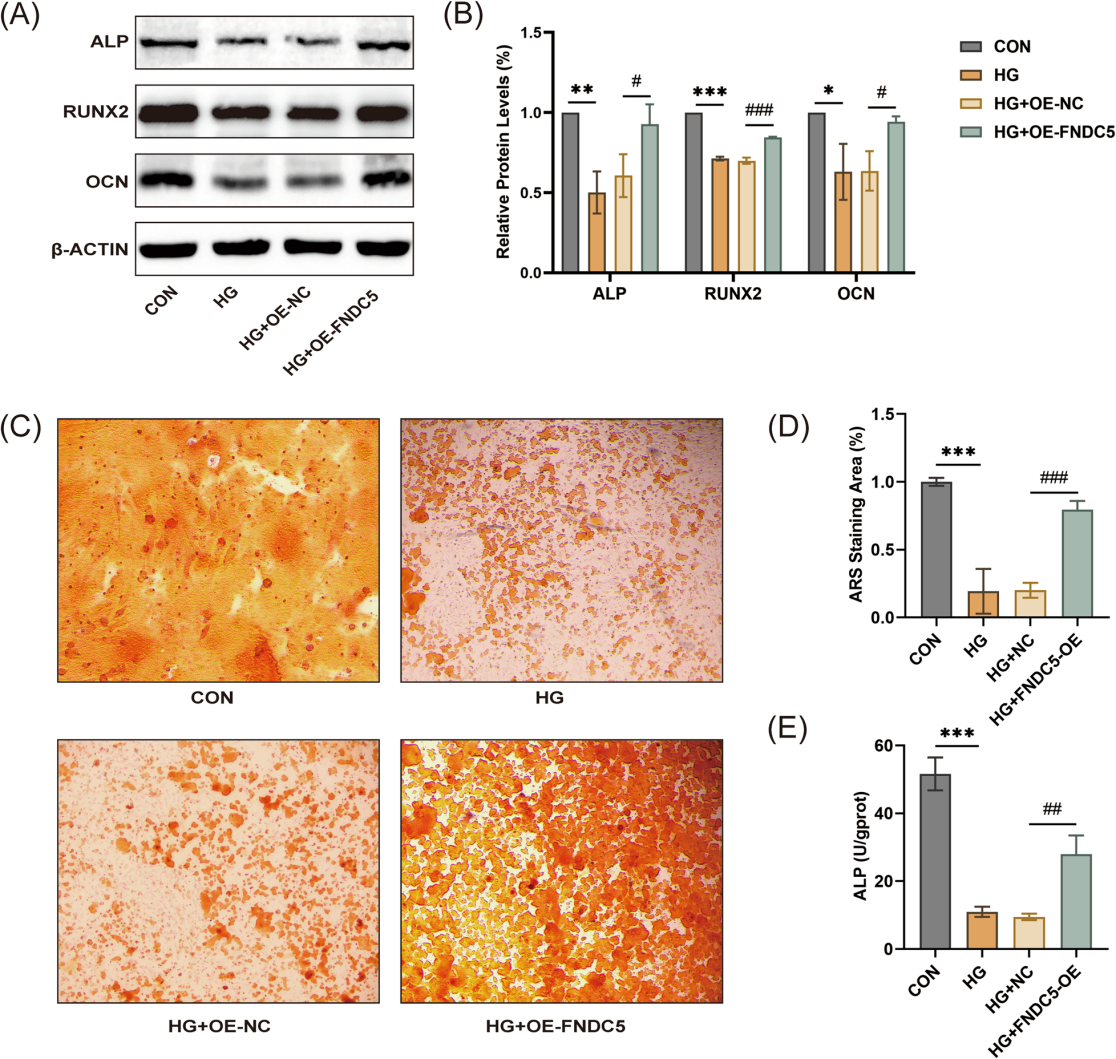
FNDC5 overexpression improved HG-induced osteogenic differentiation in MC3T3-E1 cells. (**A**, **B**) The protein levels of ALP, RUNX2, and OCN. (**C**, **D**) Representative pictures and quantitative results of Alizarin red staining. (**E**) The results of the ALP activity test. *P < 0.05, **P < 0.01, ***P < 0.001 versus the con group. ^#^*P* < , ^##^*P* < 0.01, ^###^*P* < 0.001 versus the HG group

To investigate the impact of FNDC5 overexpression on HG-induced osteogenic differentiation, we conducted further experiments. Western blot results showed that FNDC5 overexpression upregulated the expression of ALP, RUNX2, and OCN (Fig. 7A, B). Alizarin red staining showed that FNDC5 overexpression improved the production of mineralized nodules (Fig. 7C). Besides, FNDC5 overexpression also increased alkaline ALP activity (Fig. 7D). These data indicated that FNDC5 overexpression promoted the osteogenic differentiation of osteoblasts.

### FNDC5 negatively modulated ferroptosis by the eIF2α-ATF4-CHOP pathway

**Fig. 8:**
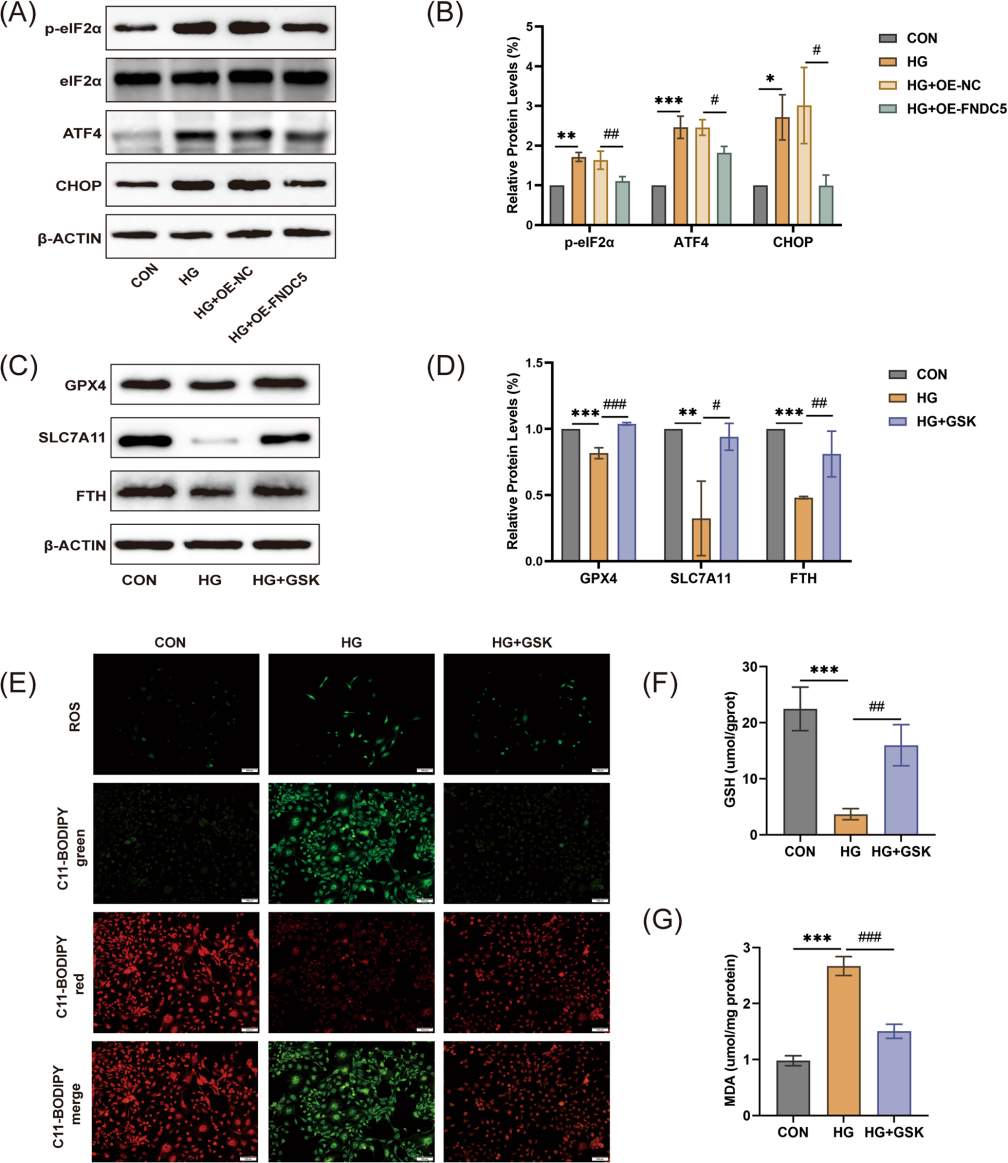
FNDC5 modulated ferroptosis by the eIF2α-ATF4-CHOP pathway. (**A**, **B**) The cells were treated with HG with or without FNDC5 overexpression. Protein levels of eif2a, ATF4, and CHOP. *P < 0.05, **P < 0.01, ***P < 0.001 versus the con group. ^#^*P* < 0.05, ^##^*P* < 0.01 versus the HG group. The cells were treated with HG with or without GSK. (**C**, **D**) Protein levels of GPX4, SLC7A11, and FTH. (**E**) Representative images of the DCFH-DA staining (scale: 100 μm) and C11-BODIPY staining (scale bar: 100 μm) images. Red images represented nonoxidized lipids, while green images represented oxidized lipids. (**F**) GSH content and (**G**) MDA levels. **P < 0.01, ***P < 0.001 versus the con group. ^#^*P* < 0.05, ^##^*P* < 0.01, ^###^*P* < 0.05 versus the HG group

Increased endoplasmic reticulum (ER) stress has been found to be associated with ferroptosis. The upregulation of p-eIF2α, ATF4, and CHOP in HG-induced MC3T3-E1 cells indicated the activation of ER stress (Fig S5A, B). Overexpression of FNDC5 inhibits the activation of the eIF2α-ATF4-CHOP pathway (Fig. 8A, B). These findings suggest that FNDC5-regulated osteoblasts ferroptosis might be mediated by the activation of the eIF2α-ATF4-CHOP signaling pathway.

Next, GSK (a selective inhibitor of PERK) was used to explore the relationship between ER stress and HG-induced ferroptosis. After treatment with GSK, the expression levels of p-eIF2α, ATF4, and CHOP were decreased (Additional file 1: Fig. S5C, D). Besides, compared to the HG group, GSK pretreatment partially restored the expression levels of GPX4, SLC7A11, and FTH (Fig. 8C, D). GSK improved the production of ROS and lipid peroxide (Fig. 8E). Moreover, GSK pretreatment significantly inhibited GSH depletion (Fig. 8F) and reduced MDA accumulation (Fig. 8G). These results indicated that ER stress might act as an upstream signaling pathway of HG-induced ferroptosis in MC3T3-E1 cells.

## Discussion

In this study, we first employed bioinformatics to screen out an FR-DEG FNDC5. Experimental verification showed that the expression of FNDC5 was decreased in bone tissues of type 1 diabetic osteopathy. Furthermore, irisin treatment or FNDC5 overexpression improved osteoblasts ferroptosis and bone loss. Moreover, FNDC5/irisin reduced ferroptosis by inhibiting the eIF2α-ATF4-CHOP signaling pathway. These findings indicated that FNDC5/irisin could be a potential therapeutic target for improving diabetic osteopathy.

Type 1 diabetic osteopathy is characterized by deterioration of bone microarchitecture, low bone mineral density, and an increased risk of fragility fractures[41]. Ferroptosis is thought to be involved in the development of diabetic osteopathy [42, 43], but the underlying mechanism remains unclear. Here, we used the FerrDb database and the GSE189112 dataset to screen out hub genes related to ferroptosis. Combining previous research and p-values of differential expression, we ultimately identified FNDC5 as the candidate hub gene. To explore the therapeutic effect of irisin in diabetic osteopathy, we constructed an STZ-induced type 1 diabetes osteopathy mouse model and treated the mice with irisin. The results of micro-CT and histopathological staining showed that irisin treatment significantly improved bone microarchitecture and bone mass. Notably, consistent with the latest research in STZ-induced diabetic rats, we found that changes in trabecular bone are more pronounced than changes in cortical bone [44]. Clinical studies have also revealed that patients with type 1 diabetes tend to have lower trabecular bone mass [45]. This phenomenon can be attributed to the higher metabolic activity of trabecular bone compared to cortical bone, leading to early onset trabecular bone loss in cases of osteoporosis.

Type 1 diabetic osteopathy is commonly attributed to a reduction in bone formation. In our study, these results are in line with previous studies that have demonstrated the ability of irisin to enhance the proliferation and differentiation of osteoblasts[19, 46]. Next, we set out to explore the potential mechanism by which FNDC5/irisin regulates ferroptosis in type 1 diabetic osteopathy. Recent studies have demonstrated that FNDC5/irisin plays a negative regulatory role in ferroptosis. In hypoxic cardiomyocytes, FNDC5/irisin inhibited ferroptosis and improved mitochondrial dysfunction by Nrf2/HO-1 pathway [47]. Additionally, irisin also could protect against sepsis-associated acute kidney injury through anti-ferroptosis via activating the SIRT1/Nrf2 axis [48]. Thus, we hypothesized that irisin plays a protective role by modulating the ferroptosis process in type 1 diabetic osteopathy. The results of our study confirmed that irisin suppressed ferroptosis of bone tissues in type 1 diabetic osteopathy. Additionally, we observed that FNDC5 overexpression also suppressed ferroptosis of HG-induced osteoblasts. This was supported by a decrease in ROS, MDA, and Fe2 + , as well as an increase in GSH. Furthermore, we observed significant changes in the expression of critical proteins, including GPX4, SLC7A11, and FTH. These findings provided evidence for the role of FNDC5/irisin in protecting against ferroptosis in type 1 diabetic osteopathy.

Subsequently, we explored the signaling pathway of FNDC5/irisin in inhibiting ferroptosis and regulating osteogenic function. ER stress is a physiological or pathological state in which misfolded proteins accumulate in the endoplasmic reticulum. Prolonged or severe ER stress can lead to cell death. Emerging evidence suggests that ER stress signaling is closely linked to ferroptosis. For instance, ER stress mediated Gα12 overexpression has been shown to facilitate ferroptosis in acute hepatic injury [49]. Similarly, in bronchial epithelial cells, ferroptosis was triggered by whole cigarette condensate via ER stress [50]. However, the association between ferroptosis and ER stress has not been described in osteoporosis. In our study. inhibiting ER stress pathways significantly reduced ferroptosis in MC3T3-E1 cells treated with HG. The results indicated that eIF2α/ATF4/CHOP is the upstream of ferroptosis. Moreover, overexpression of the FNDC5 strikingly inhibited ER stress by reducing the expressions of the eIFα, ATF4, and CHOP, indicating the eIFα-ATF4-CHOP pathway as the downstream of FNDC5. Previous studies have also highlighted the role of FNDC5 in regulating ER stress [51, 52]. Overall, our findings indicated that overexpression of FNDC5 suppressed ferroptosis in HG-induced MC3T3-E1 cells through the eIF2α-ATF4-CHOP pathway.

In conclusion, this study is the first to combine the FerrDb database with a type 1 diabetic osteopathy-related GEO dataset. Through bioinformatic analysis, we identified FNDC5 as an essential FR-DEG. Our study demonstrated that FNDC5 overexpression or irisin treatment significantly suppressed ferroptosis and promoted osteogenesis in type 1 diabetic mice through the eIF2α-ATF4-CHOP pathway. The study explored a new regulatory pathway of FNDC5/irisin in osteoporosis, expanded the research direction of FNDC5/irisin in bone metabolism diseases, and provided a possible new target for type 1 diabetic osteopathy. The mechanisms underlying the downregulation of FNDC5 expression in osteoblasts and the specific molecular mechanisms by which FNDC5 inhibits the eIF2α-ATF4-CHOP pathway in type 1 diabetic osteopathy deserve further exploration in the future.

### Supplementary Information

**Additional file 1**. FNDC5/irisin ameliorates bone loss of type 1 diabetes by suppressing endoplasmic reticulum stress‑mediated ferroptosis.

## Data Availability

Publicly available datasets were analyzed in this study. These data can be found at the following URL: GSE189112 dataset (https://www.ncbi.nlm.nih.gov/geo/query/acc.cgi?acc=GSE189112), and FerrDb online database (http://www.zhounan.org/ferrdb/).

## Abbreviations

T1DM: Type 1 diabetes mellitus
GPX4: Glutathione peroxidase 4
GSH: Glutathione
ER: Endoplasmic reticulum
eIF2α: Eukaryotic initiation factor 2 alpha
CHOP: C/EBP-homologous protein
GEO: Gene Expression Omnibus
GO: Gene Ontology
PPI: Protein-protein interaction
STZ: Streptozotocin
Ct. BMD: Cortical bone mineral density
BV/TV: Bone volume fraction
Tb. Th: Trabecular thickness
PINP: Procollagen I N-terminal propeptide
GSK: GSK2606414
ECL: Super electrochemiluminescence
SLC7A11: Solute carrier family 7 member 11
FTH: Ferritin heavy chain
TEM: Transmission Electron Microscope
ROS: Reactive oxygen species
Xc- system: Cystine-glutamate antiporter system
FNDC5: Fibronectin type III domain-containing protein 5
PERK: Protein kinase-like kinase
ATF4: Activated transcription factor 4
FR-DEG: Ferroptosis-related differential gene
DEGs: Differentially expressed genes
KEGG: Kyoto Encyclopedia of Genes and Genomes
SPF: Special pathogen-free
FBG: Fasting blood glucose
Tb. BMD: Trabecular bone mineral density
Tb. N: Trabecular number
Tb. Sp: Trabecular separation
OCN: Osteocalcin
RT-qPCR: Real-time quantitative polymerase chain reaction
ALP: Alkaline phosphatase
RUNX2: Runt-related transcription factor 2
MDA: Malondialdehyde
HG: High glucose
