# Supplementary material for: FNDC5/irisin ameliorates bone loss of type 1 diabetes by suppressing endoplasmic reticulum stress‑mediated ferroptosis
Source: J Orthop Surg Res. 2024 Mar 30;19:205. doi: 10.1186/s13018-024-04701-3 (PMC10981808; doi:10.1186/s13018-024-04701-3)
Supplement: Supplementary file 1 — Additional file 1. FNDC5/irisin ameliorates bone loss of type 1 diabetes by suppressing endoplasmic reticulum stress‑mediated ferroptosis. [file 13018_2024_4701_MOESM1_ESM.docx]

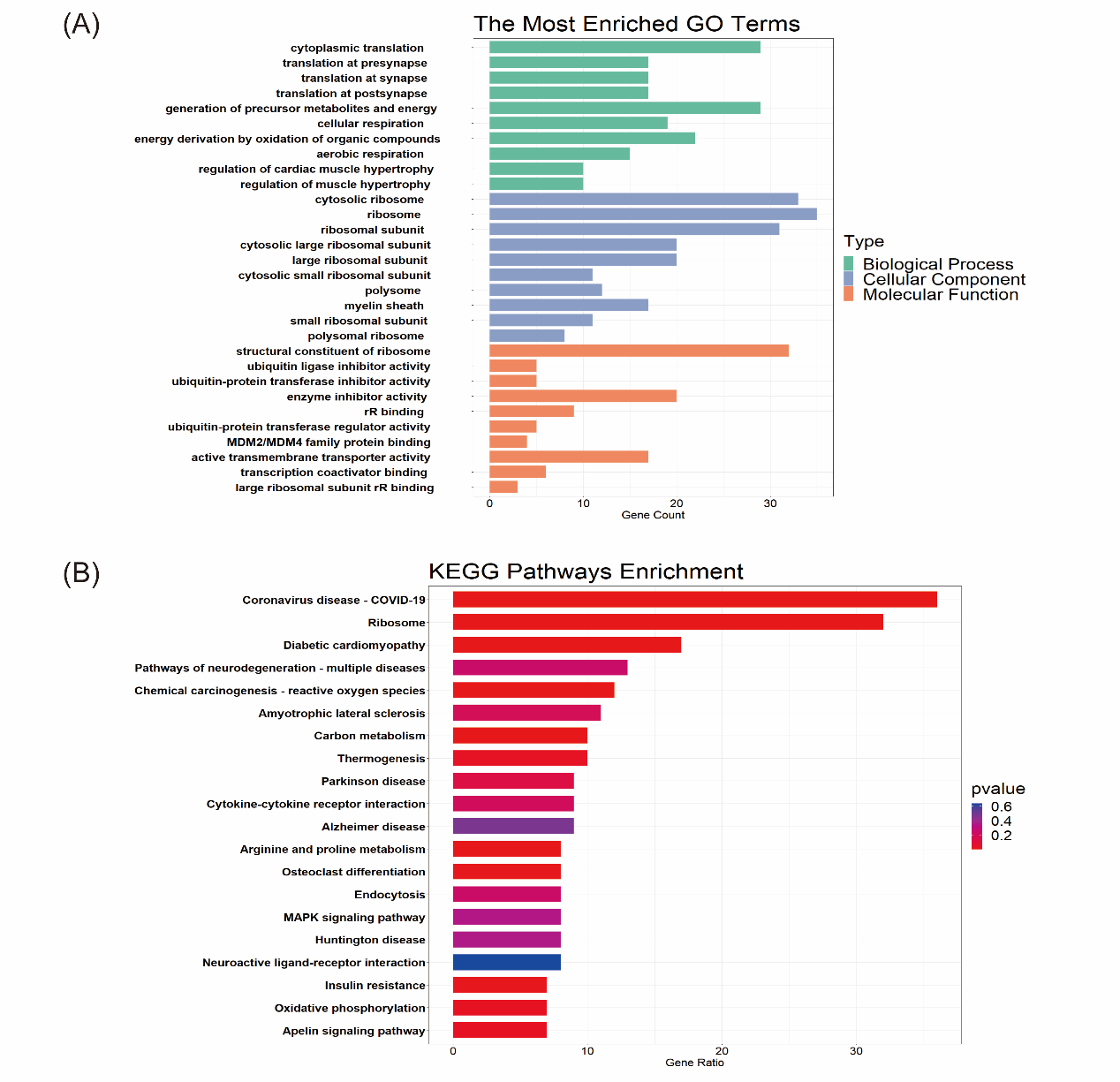


**Fig.S1 GO and KEGG enrichment analysis of DEGs.** (**A**) The top 10 GO terms of biological processes, cellular components, and molecular functions. (**B**) KEGG enrichment analysis of DEGs.


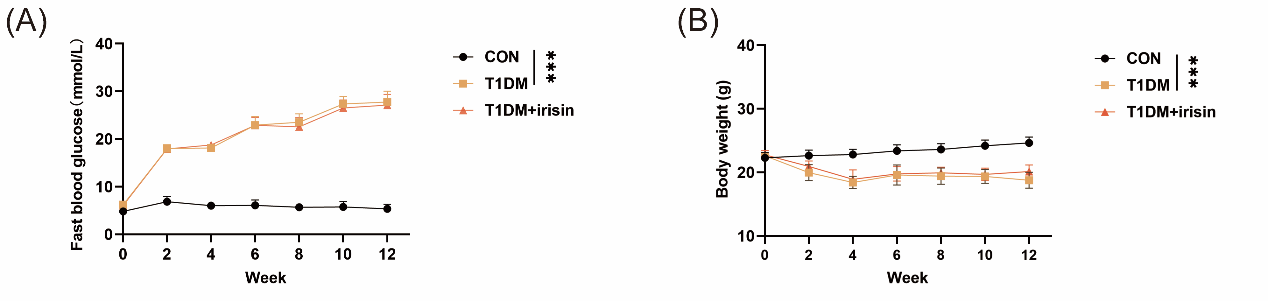


**Fig.S2 The fasting blood glucose(A)** **and body weight (B) were measured every 2 weeks.** ****P*< 0.001 vs. the con group.

**
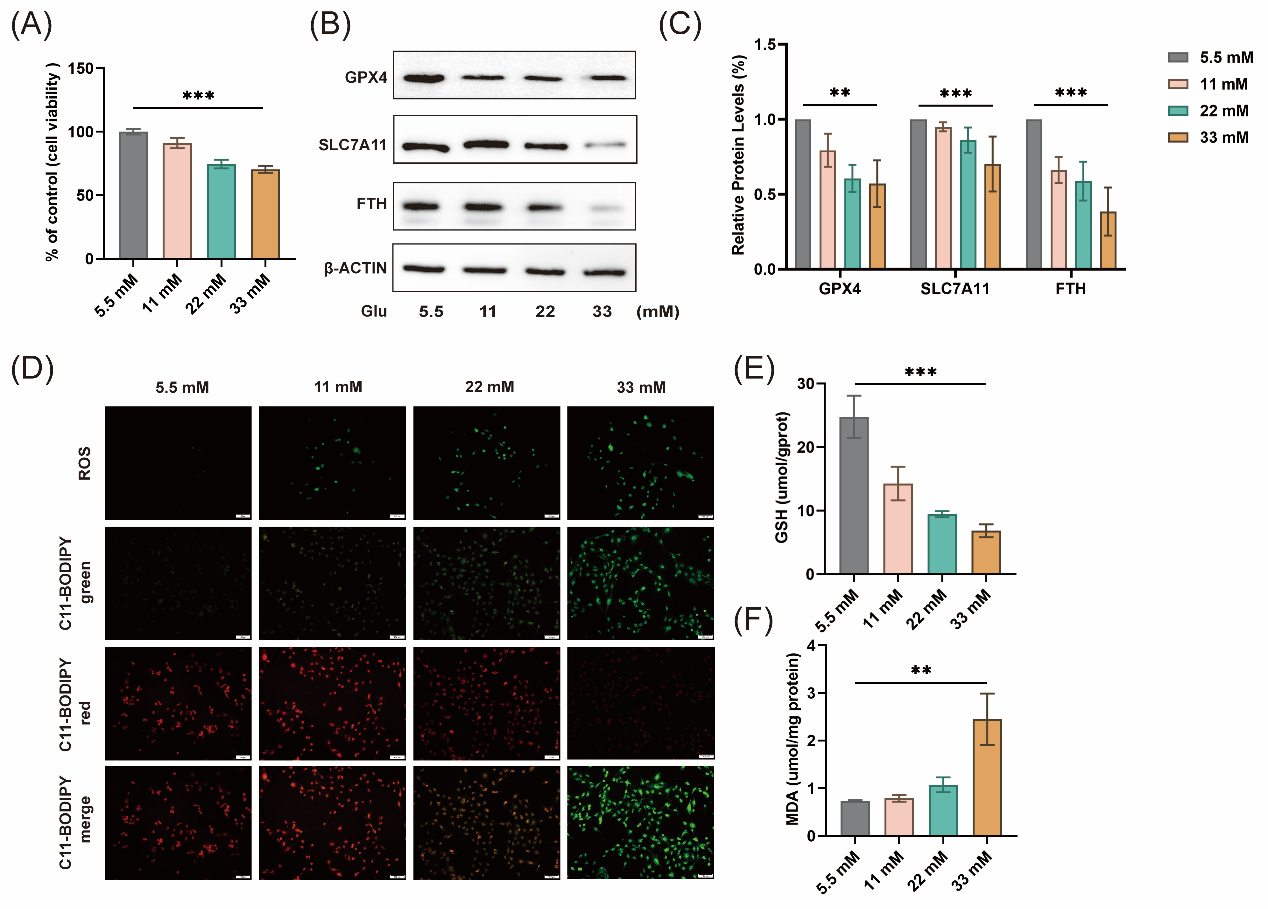
Fig.S3** **High glucose (HG) activated the ferroptosis of MCST3-E1 cells.** MC3T3-E1 cells were cultured with various glucose concentrations (5.5, 11, 22, or 33 mM). (**A**) Cell viability was measured using CCK8. (**B-C**) Relative protein levels of GPX4, SLC7A11, and FTH. (**D**) Representative images of the DCFH-DA staining (scale: 100μm) and C11-BODIPY staining (scale bar: 100μm) images. Red images represented nonoxidized lipids, while green images represented oxidized lipids. (**E**) GSH levels and (**F**) MDA contents. **P* < 0.05, ***P*< 0.01, ****P* < 0.001 vs. the 5.5mm group.


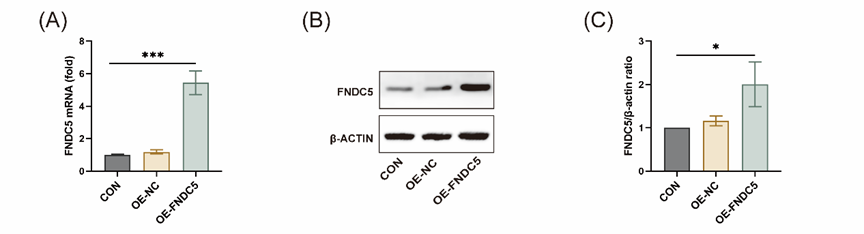


**Fig.S4Transfection efficiency was verified after transfection of the FNDC5 overexpression plasmid.** (**A**) Relative mRNA expression of FNDC5. (**B-C**) Relative protein level of FNDC5. OE-NC means negative control, and OE-FNDC5 means overexpression of FNDC5. **P* < 0.05, ****P* < 0.001 vs. the con group.

**
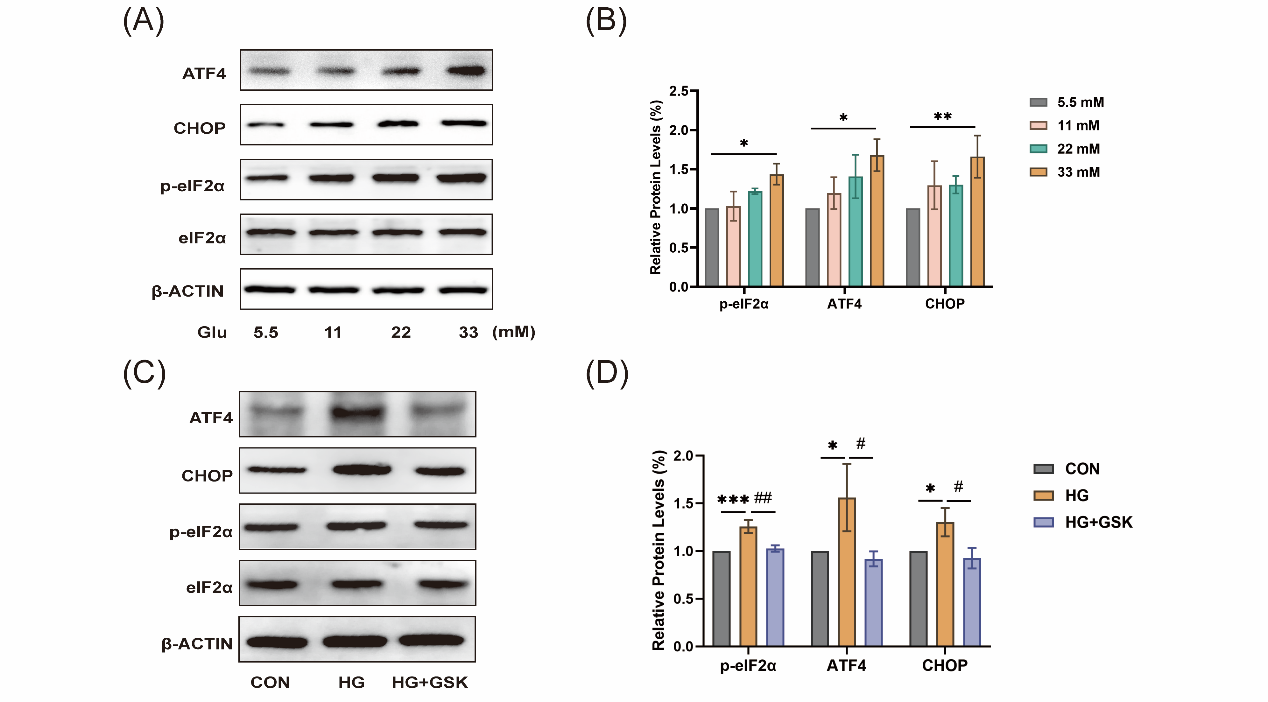
**

**Fig.S5** **HG activated the eIF2α-ATF4-CHOP pathway and GSK treatment inhibited the eIF2α-ATF4-CHOP pathway.** (**A-B**) Relative protein levels of p-eIF2a, ATF4, and CHOP in MC3T3-E1 cells cultured with various glucose concentrations (5.5, 11, 22, or 33 mM). **P* < 0.05, ***P* < 0.01, ****P* < 0.001 vs. the 5.5mm group. (**C-D**) Relative protein levels s of p-eIF2a, ATF4, and CHOP in MC3T3-E1 cells treated with or without GSK. **P* < 0.05, ****P* < 0.001 vs. the con group. ^#^*P* < 0.05, ^##^*P* < 0.01 vs. the HG group.
